# Novel 2D/3D Hybrid Organoid System for High-Throughput Drug Screening in iPSC Cardiomyocytes

**DOI:** 10.3390/therapeutics2030011

**Published:** 2025-06-27

**Authors:** Jordann Lewis, Basil Yaseen, Haodi Wu, Anita Saraf

**Affiliations:** 1 Heart Institute, UPMC Children’s Hospital of Pittsburgh, Pittsburgh, PA 15224, USA; 2 UPMC Heart and Vascular Institute, University of Pittsburgh Schools of the Health Sciences, Pittsburgh, PA 15213, USA; 3 Division of Cardiology, Department of Medicine, University of Pittsburgh, Pittsburgh, PA 15213, USA

**Keywords:** iPSC, doxorubicin, cardiotoxicity, cardiomyocytes, drug screening, organoid

## Abstract

**Background:**

Human induced pluripotent stem cell cardiomyocytes (hiPSC-CMs) allow for high-throughput evaluation of cardiomyocyte (CM) physiology in health and disease. While multimodality testing provides a large breadth of information related to electrophysiology, contractility, and intracellular signaling in small populations of iPSC-CMs, current technologies for analyzing these parameters are expensive and resource-intensive.

**Methods:**

We have designed a novel 2D/3D hybrid organoid system that can harness optical imaging techniques to assess electromechanical properties and calcium dynamics across CMs in a high-throughput manner. We validated our methods using a doxorubicin-based system, as the drug has well-characterized cardiotoxic, pro-arrhythmic effects.

**Results:**

This novel hybrid system provides the functional benefit of 3D organoids while minimizing optical interference from multilayered cellular systems through our cell-culture techniques that propagate organoids outwards into 2D iPSC-CM sheets. The organoids recapitulate contractile forces that are more robust in 3D structures and connectivity, while 2D CMs facilitate analysis at an individual cellular level, which recreated numerous doxorubicin-induced electrophysiologic and propagation abnormalities.

**Conclusions:**

Thus, we have developed a novel 2D/3D hybrid organoid model that employs an integrated optical analysis platform to provide a reliable high-throughput method for studying cardiotoxicity, providing valuable data on calcium, contractility, and signal propagation.

## 1. Introduction

Induced pluripotent stem cell-derived cardiomyocytes (iPSC-CMs) are being widely used for disease and toxicity modeling and as a preliminary screening tool for drug discovery [1–3]. The greater appeal of iPSC-derived cells as a drug discovery tool is that these cells are patient-derived, thereby providing immediate clinical relevance; can be differentiated into multiple cell types, which preserve the genetic background of the disease under study; and can be generated in large quantities in a relatively small amount of time. Hence, iPSCs have replaced previous cellular models for drug discovery, including immortalized cell lines, embryonic stem cells (ESCs), and animal models where disease physiology cannot be faithfully recreated. Cardiomyocytes derived from iPSCs have a greater advantage as they reflect cellular morphology as well as electrical and mechanical properties that are central to their function. Hence, evaluating the toxicity profile of drugs using iPSC-CMs provides a wealth of information beyond cell death, including the incidence of arrhythmia and conduction of calcium and action potentials across multiple cells. The technology to detect these functional characteristics currently relies on specialized and expensive instrumentation such as microelectrode arrays (MEAs), which capture electrical activity using microscopic electrodes in each well of a plate.

Thus, there is a need for an improved, high-throughput drug screening method that can distinguish effects on cardiac tissue. Current drug screening methods utilize 2D cell sheets and a rapid and relatively simple model to study CM structure and function. This does not emulate cardiac tissue, with the multitude of cell–cell interactions [4]. In recent years, three-dimensional (3D) organoid models have helped overcome the limitations of 2D cell culture, as they display more mature phenotypes, better reflect cell morphology, and recapitulate cell–cell communication networks [5].

However, it remains difficult to screen drug toxicity in 3D cell cultures due to current systems being optimized for 2D monolayered systems. For example, the patch-clamp technique relies on the isolation of individual cells, which can be difficult to obtain from 3D spheroidal cell aggregates. Additionally, MEAs assess cells lying on the electrode, and thus are well-suited for 2D monolayers of cells, but contact with 3D spheroidal cell aggregates may be minimal. As an alternative technique, optical analysis of calcium transients, which correspond to action potentials, is generally accepted as an additional method for electromechanical assessment. However, 3D spheroidal organoids may still pose challenges in analysis due to optical signal interference from overlapping cells.

Therefore, we sought to design a novel 2D/3D hybrid organoid system relying on optical techniques and spatial segmentation to obtain electromechanical information on calcium dynamics, cardiomyocyte function, and signal propagation in a high-throughput manner that retains the complexity of a 3D organoid, while also allowing for isolation of single cells. We validated the efficacy of these methods by using a doxorubicin (Dox) treatment model, which has previously been extensively characterized with respect to its toxicity on cardiomyocytes and known to have cardiotoxic, pro-arrhythmic effects [6]. Furthermore, we utilized machine-learning methods to facilitate analysis of the data extracted from our model.

## 2. Methods

### 2.1. Cell Culture and Model Development

Wild-type hiPSCs were obtained from the NIH iPSC repository (ND2.0, NIH CRM control iPSC line) and plated on 6-well tissue culture plates, coated with Matrigel (Corning, NY, USA) until they reached 80–90% confluence, at which point they were split and transferred to a 6-well plate. iPSCs were differentiated using 2 mL RPMI with B27 minus (Sigma Aldrich, St. Louis, MO, USA) and 6 μM CHIR (Selleck Chemical, Houston, TX, USA) for 2 days. At 96 h, the media was replaced with RPMI B27 minus (Sigma Aldrich, St. Louis, MO, USA) and 5 μM IWR-1 (Sigma Aldrich, St. Louis, MO, USA). After 48 h, they received RPMI B27 minus, which was replaced by RPMI B27 (Sigma Aldrich, St. Louis, MO, USA) plus after an additional 48 h. At day 7, cells were transferred to an AggreWell plate (Agilent, Santa Clara, CA, USA) to generate organoids. After cells were added to the AggreWell plate, the plate was centrifuged and incubated at 37 °C for 48 h. Subsequently, organoids were dislodged from the AggreWell plate, transferred into non-adherent plates, and maintained in RPMI B27 media. Organoids were treated with 0.5 μM Dox or control media for 24 h and then switched to maintenance media with RPMI B27 for 10 days following drug treatment. At that point, organoids were placed in Ibidi wells (Ibidi, WI, USA) with Matrigel to allow for single cells to dissociate from the organoid for further evaluation. The cells were then stained with Rhod-2 (Thermofisher Scientific, Waltham, MA, USA) for calcium analysis. A 2.5 mM stock of Rhod-2 was diluted 1:1000 in RPMI B27 plus, added to cells, and incubated at 37 °C for 15–25 min, after which the cells were washed two times with RPMI B27 plus.

### 2.2. Image Capture and Video Analysis

Fifteen-second videos of unpaced, spontaneously beating cardiomyocyte organoids were captured at 20× using confocal microscopy (Nikon AIR HD camera and NIS elements AR 5.41.02 64-bit software, Melville, NY, USA) with absorbance and emission fluorescence staining. To visualize calcium transients within cells, the calcium indicator Rhod-2 was used, and then cells were stained with Cy3. Fluorophores were excited at and imaged at appropriate emission wavelengths (590 nm, red). Brightfield videos were also consecutively recorded at 20× for fifteen seconds. Videos were visually assessed for adequate distribution of 2D cells around the organoid. Videos that lacked surrounding cells or fluorescence were excluded from analysis, resulting in a total of 18 (10 control, 8 Dox) videos analyzed. Videos were uploaded into ImageJ 1.53v (NIH, Bethesda, MD, USA) and the “ROI Manager” tool was utilized to select contracting cells within each brightfield video and discrete areas of Rhod-2 excitation/emission within each fluorescent video, with an average of approximately 65 ROIs/cells identified per video.

### 2.3. Calcium Analysis

To assess calcium transients, we utilized Spiky (1.0v), an ImageJ plugin, which is a software tool that quantifies excitation–contraction related experimental data, including intracellular calcium transients [7]. It detects peaks in the input by characterizing the temporal shift from baseline of a signal in an image and then computes a variety of parameters for each peak, such as amplitude, time to peak, time between peaks, peak width, maximum slope of peak, and area under the curve. Spiky was run for each individual ROI. Default settings were used for peak detection. Full width and right and left half widths at 20% maximum represented total flux, efflux, and influx. Area under the curve is a function of fluorescence amplitude and time and thus is representative of relative calcium signal magnitude. Drift removal was applied to all outputs to normalize baseline shifts. Graphical results were evaluated for quality control (correct detection of calcium dynamic peaks).

### 2.4. Contractility Analysis

To further evaluate our model’s reliability, we analyzed brightfield videos to assess contractility, which should mirror the effects seen in calcium transient analysis based on excitation–contraction coupling. To analyze contractility, we utilized an ImageJ macro called Myocyter v-1.1.20 [8]. This tool characterizes contracting regions using two parameters: “speed”, which is the difference between consecutive frames of a video and indicates the speed of a contraction, and “amplitude”, which is the difference between the current frame and an automatically determined reference frame of the cell in its resting phase and indicates the amount of deformation of the contracting cell compared to the reference frame. The difference between the images is then calculated and presented as a plot and numerical output, generating values for mean frequency, amplitude, systole (contraction time), diastole (relaxation time), peak times (total length of contraction and relaxation), and beat time (peak-to-peak time). Using dynamic thresholding, the program precisely tracks amplitudes even when the baseline shifts. Myocyter was run using the manual ROI list and default settings for intensity threshold, particle size, peak recognition, and sensitivity of maxima and minima detection. Analysis was performed on a per-cell basis, and graphical results were evaluated for quality control (correct detection of contractile motion).

### 2.5. Regional/Spatial Segmentation

To analyze spatial differences in calcium transient and contractility parameters based on the distance of 2D surrounding cells from the beating organoid, we segmented images into regions of equally increasing distance from the beating organoid. Three concentric circles centered on the organoid were overlaid on each frame, each with a distance of 185 microns between. This distance was selected to have three equally spaced regions surrounding the organoid that fit within the image frame and reflected a proximal-to-distal spatial gradient to compare cells that were in the immediate surroundings of the organoid with those that had migrated a further distance. Preliminary analysis also showed significant differences in conduction with Dox treatment at this distance. The regions were labeled “Center” for the organoid, “Close” for the first adjacent region, “Mid/Intermediate” for the second region from the center, and “Far” represents the remaining area in the frame of the image.

### 2.6. Machine Learning

First, the evaluation of calcium transient abnormality was conducted by two experts for the signal from each cell. In instances of discordance between the two experts, a third expert inspected the signal to resolve discrepancies. A cell was labeled as having abnormal calcium transients if it had substantial atypical calcium transient morphology, such as bifid or multi-spiked peaks, plateauing of calcium signal during uptake, increasing calcium during uptake as indicated by a second upstroke occurring prior to complete signal decay, and evidence of spontaneous calcium release between peaks. Next, parameters obtained from Spiky were used to train the SVM. To ensure our model was not overfitting data, we used 10-fold cross-validation. Additionally, we tuned the regularization hyperparameters of the classifier model, denoted by *C*, using a grid search within a range of 0.001–1000. To assess the performance of the model, we relied on sensitivity, specificity, and accuracy calculations, as well as the area under the ROC curve.

### 2.7. Statistical Analysis

Statistical analyses were performed in GraphPad Prism 10.2.1 and Python 3.8.17. All data are reported as mean ± standard error of the mean unless noted otherwise. Two-tailed Student’s *t*-tests were performed to compare 2D and 3D cells and to compare control and Dox-treated groups. One-way ANOVA followed by Tukey’s post-hoc test was used to detect differences across spatial regions. A *p*-value of less than 0.05 was considered statistically significant.

## 3. Results

### 3.1. Hybrid 2D/3D Models Can Overcome Challenges Associated with Traditional 2D Sheets and3D Organoids

**Figure 1.**
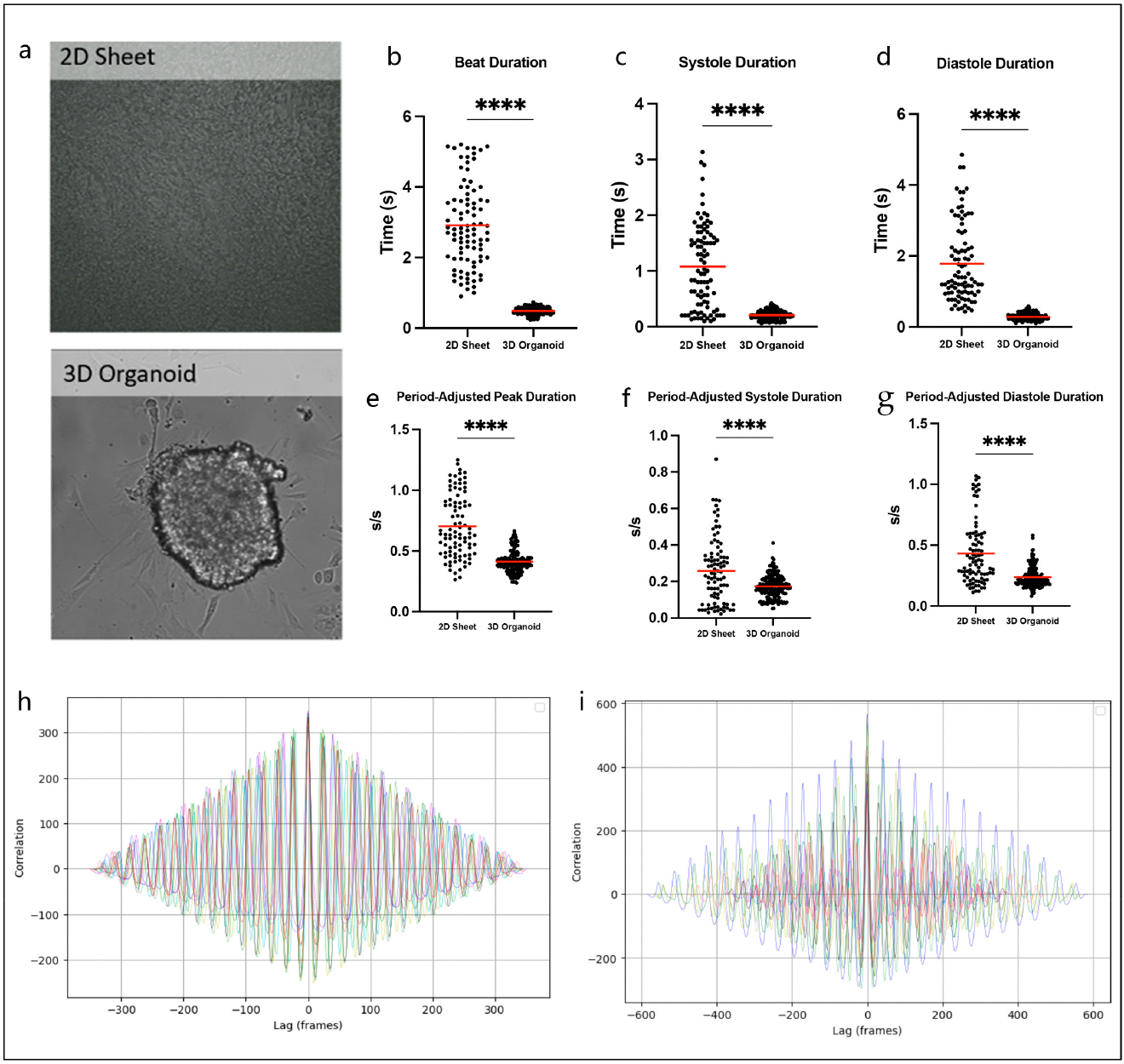
(**a**) Representative brightfield videos of traditional 2D cellular sheet, 3D organoid and a hybrid 3D/2D model, can see in supplementary files. (**b**–**d**) Total beat duration, systole duration, and diastole duration are prolonged in 2D sheets (n = 91–171). Notably, 2D sheets have visibly greater ranges for these durations, indicating less synchronicity, while cells within 3D organoids have a more compact distribution, providing evidence of improved connectivity. (**e**–**g**) Prolonged duration and increased variability amongst beat, systole, and diastole durations remains even when controlling for overall contraction time and wavelength period (n = 91–171). Cumulative overlays of cross correlation analysis for contractility (**h**) and fluorescence (**i**) for each individual video (n = 10). This analysis quantifies of how similar the signal originating from the 2D cells is to the signal from the 3D organoids in our hybrid model within each video. The strong peak at Lag = 0 indicates the signals are highly synchronous. Two-tailed *t*-test was used for all statistical comparisons **** *p* < 0.0001).

To provide justification for the development of this novel model, we first analyzed traditional 2D sheets and 3D organoids to elucidate the associated challenges. By comparing 2D cellular sheets and 3D organoids (Figure 1a), we demonstrated that under the same conditions, 2D sheets and 3D organoids behave differently. 2D sheets have prolonged total beat duration (2.91 ± 0.124 s vs. 0.4881 ± 0.008 s, *p* < 0.0001, Figure 1b), systole duration (1.077 ± 0.079 s vs. 0.207 ± 0.006 s, *p* < 0.0001, Figure 1c), and diastole duration (1.782 ± 0.114 s vs. 0.281 ± 0.007 s, *p* < 0.0001, Figure 1d), and this remains true even after adjusting for overall contraction time and wavelength period (peak: 0.703 ± 0.027 s/s vs. 0.411 ± 0.007 s/s, *p* < 0.0001; systole: 0.258 ± 0.019 s/s vs. 0.173 ± 0.005 s/s, *p* < 0.0001; diastole: 0.433 ± 0.027 s/s vs. 0.238 ± 0.006 s/s, *p* < 0.0001, Figure 1e–g). Additionally, it is evident that cells across 2D sheets do not beat as synchronously, given the larger variability of times for total beat, systole, and diastole duration. To demonstrate that our model overcomes such challenges, we performed a cross-correlation analysis to quantify the degree of association between our 2D and 3D cell signals in our hybrid model. This allowed us to compare the waveforms for contractility amplitude and displacement in brightfield images (Figure 1h) as well as the waveforms for calcium fluorescence intensity (Figure 1i). Because the maximum correlation is at lag time zero for each video under both conditions, we can conclude that there is no significant time delay between the 2D cells of our hybrid model and the 3D organoid, which confirms they beat synchronously and are identical and interconnected.

### 3.2. hiPSC-CMs Demonstrate Cardiotoxic Effects of Doxorubicin

**Figure 2.**
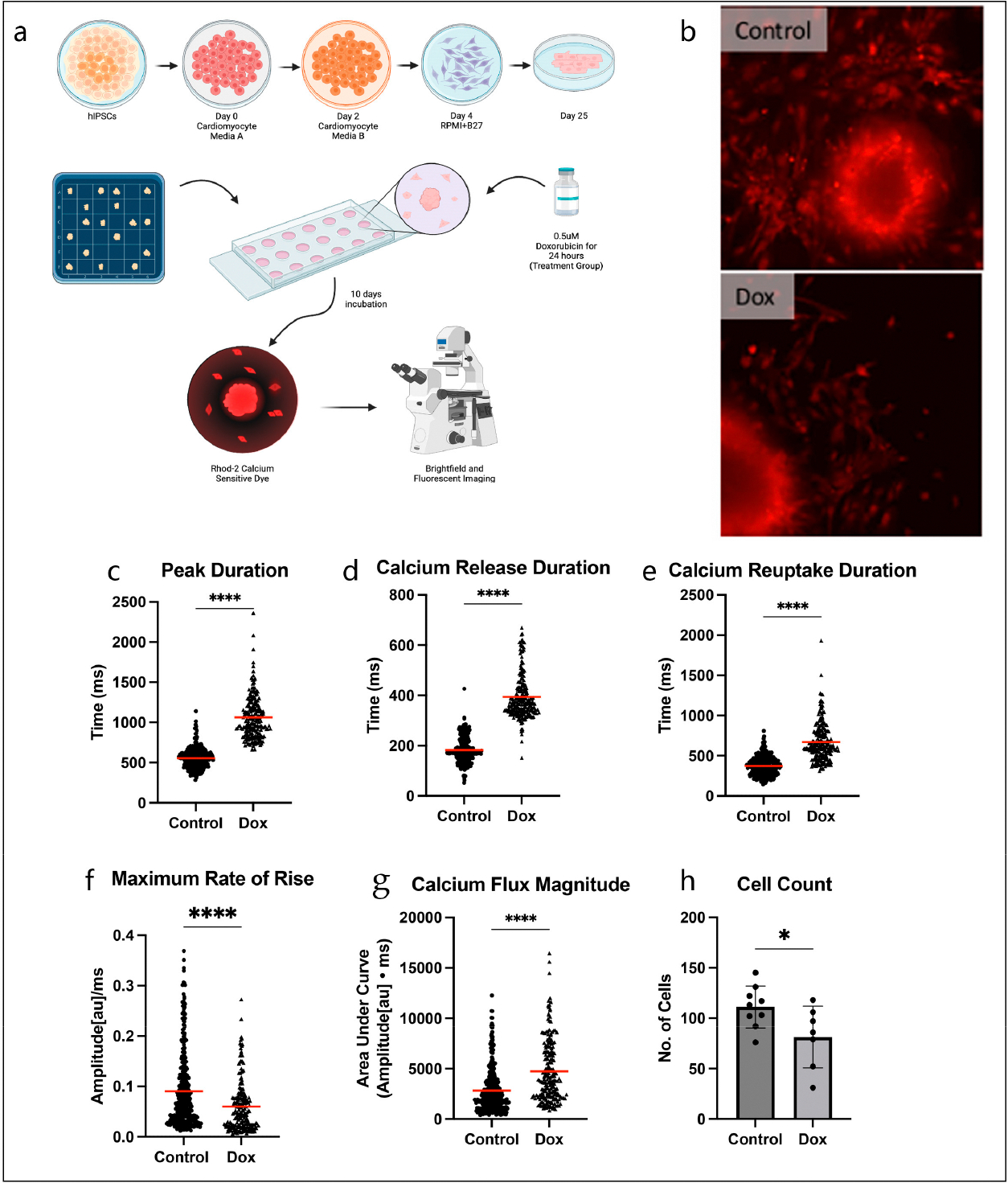
(**a**) Schematic of experiment. (**b**) Representative videos of control and Dox organoids and cells with calcium-sensitive Rhod-2 dye, can see in supplementary files. (**c**) Calcium transient peak duration is prolonged in Dox group (Unpaired *t*-test, n = 209–342). (**d**) Calcium release duration is prolonged in Dox group (Unpaired *t*-test, n = 209–342). (**e**) Calcium reuptake duration is prolonged in Dox group (Unpaired *t*-test, n = 209–342). (**f**) Maximum rate of rise for calcium transient peak is lower in Dox group (Unpaired *t*-test, n = 209–342). (**g**) Area under curve for calcium transient peak, which is indicative of magnitude of calcium, is greater in Dox group (Unpaired *t*-test, n = 209–342). (**h**) The number of cells around the organoid is decreased in Dox group (Unpaired *t*-test, n = 7–9). (* *p* < 0.05, **** *p* < 0.0001).

The cardiotoxic effects of Dox on hiPSC-CMs have been well-established, particularly in regard to abnormal calcium handling [6]. First, we reproduced these findings to confirm that Dox had accurately affected the treatment group. We used the calcium marker Rhod-2 and recorded fluorescence in our model to analyze calcium dynamics (Figure 2a,b). Our optical analysis of calcium transient images revealed that Dox-treated cells had reduced fluorescence peak maximum rate of rise (0.090 ± 0.004 a.u./ms vs. 0.060 ± 0.003 a.u./ms, *p* < 0.0001) and prolonged peak duration (556.2 ± 6.72 ms vs. 1063 ± 17.9 ms, *p* < 0.0001), with longer times for both calcium release (182.1 ± 2.74 ms vs. 393.6 ± 6.13 ms, *p* < 0.0001) and calcium reuptake (374.1 ± 6.19 ms vs. 669.6 ± 16.22 ms, *p* < 0.0001) compared to controls (Figure 2c–f), as has been previously reported [9,10]. Dox-treated cells also had a greater area under the curve, corresponding to higher levels of cytosolic calcium compared to controls (2823 ± 119.2 a.u.*ms vs. 4745 ± 212.9 a.u.*ms, *p* < 0.0001), which is also consistent with previous reports of Dox-induced calcium overload (Figure 2g) [11]. In alignment with Dox’s pro-apoptotic effects on viability [12], the Dox group had a decreased spread of cells, demonstrated by significantly fewer total surrounding cells per organoid (111.2 ± 6.92 vs. 81.29 ± 11.57, *p* = 0.0350, Figure 2h).

### 3.3. 2D/3D Hybrid Organoid Model Displays Signal Propagation Effects

**Figure 3.**
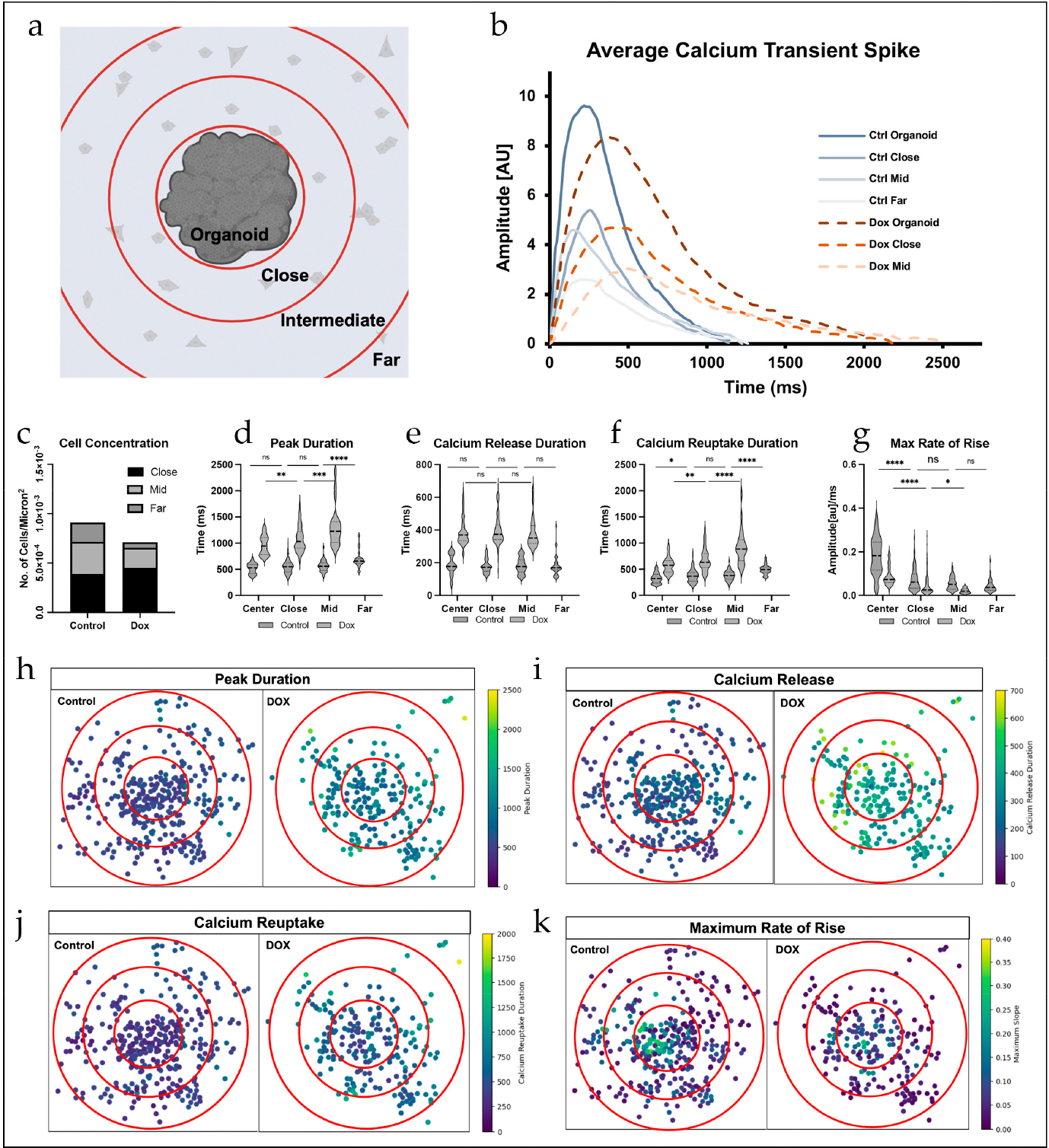
(**a**) Schematic of spatial segmentation to assess regional differences. (**b**) Representative calcium transient peak waveforms for each group. (**c**) Cellular concentration decreased in the further regions in Dox group. (**d**) Peak duration prolonged with increased distance from the organoid in Dox group (One-way ANOVA with Tukey post-hoc test, n = 23–144). (**e**) Calcium release duration was stable across regions in both groups (One-way ANOVA with Tukey post-hoc test, n = 23–144). (**f**) Calcium reuptake duration prolonged with increased distance from the organoid in Dox group (One-way ANOVA with Tukey post-hoc test, n = 23–144). (**g**) Maximum rate of rise decreased with increased distance from organoid in Dox group. In control group, it only decreased from organoid to first adjacent “Close” region (One-way ANOVA with Tukey post-hoc test, n = 23–144). (**h**–**k**) Heat maps of parameters analyzed in (**d**–**g**). Each dot represents an individual ROI/cell that was analyzed. The center of the graphs was normalized to the center point of each organoid. (* *p* < 0.05, ** *p* < 0.01, *** *p* < 0.001, **** *p* < 0.0001, ns = non-specific).

To assess calcium signal propagation originating from the organoid, we segmented the area surrounding the organoid and analyzed the same parameters that were used to evaluate overall Dox cardiotoxicity within each region (Center = Organoid, Close = 1st adjacent, Mid/Intermediate = 2nd adjacent, and Far = remaining outlying region, Figure 3a). Again, the Dox group exhibited decreased propagation of cells, with reduced cellular concentration within each region further from the organoid (Figure 3b). In general, 2D Dox-treated cells had wider waveforms, with diminished peaks with gradual slopes compared to controls, and these differences amplified as distance from the organoid increased (Figure 3c). Dox-treated cells had significant prolongation of calcium transient duration as the distance from the center increased (Center vs. Close: *p* = 0.0036, Close vs. Mid: *p* = 0.0001), while control cells displayed an increased peak duration only in the farthest region (*p* < 0.0001) (Figure 3d). Similarly, the duration of calcium uptake increased in a stepwise manner in Dox-treated cells (Center vs. Close: *p* = 0.0025, Close vs. Mid: *p* < 0.0001). Calcium uptake duration also increased in control cells but was only significant between Center and Close (*p* = 0.0462) and Mid compared to Far (*p* < 0.0001) (Figure 3f). In both groups, the duration of calcium release did not change across regions (Figure 3e), but the maximum rate of rise for calcium release was affected. It was significantly reduced between regions for Dox-treated cells (Center vs. Close: *p* < 0.0001, Close vs. Mid: *p* = 0.0258), while in control cells, the only significant decrease across adjacent regions was between the organoid and the close region (*p* < 0.0001) (Figure 3g). Spatial alterations of these parameters (calcium peak duration, release duration, reuptake duration, and maximum rate of rise) on a per-cell basis are visualized in Figure 3h–k. Despite signal duration changes on a millisecond scale in both control and Dox-treated organoids and cells, calcium fluorescence was grossly synchronized among Center, Close, Intermediate, and Far regions, indicating that the 2D cells retained connection to the 3D organoid and its properties, which provided validation to our hybrid model and methodology (Supplemental Videos S1 and S2).

### 3.4. Machine Learning to Identify Abnormal Calcium Transients

**Figure 4.**
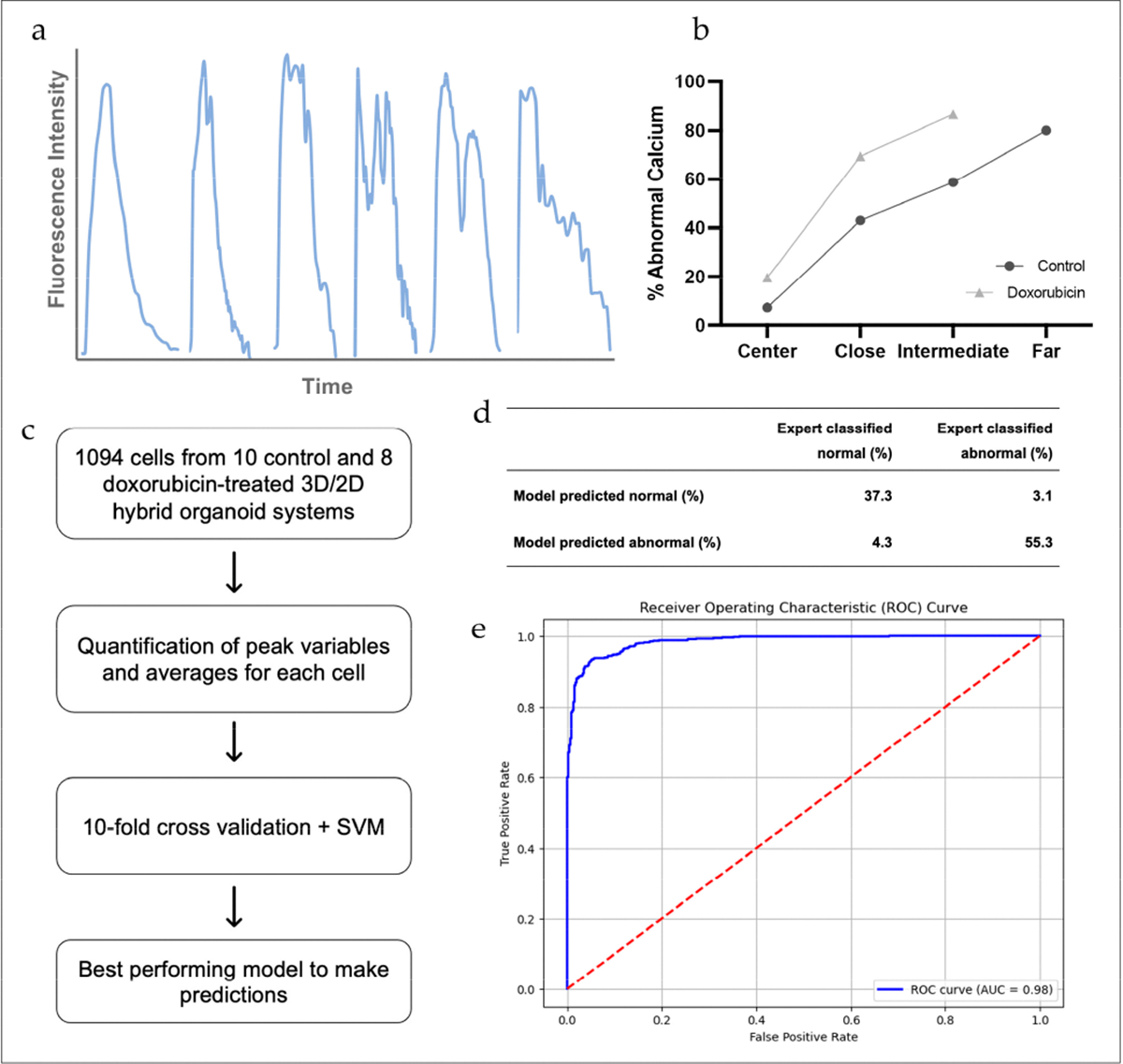
(**a**) The first waveform is an example of normal calcium transient morphology. The remaining waveforms are examples of abnormal calcium transient morphology that were observed. (**b**) Both the control and Dox groups had abnormal calcium transients, and the proportion of cells with abnormal calcium transients increased with further distance from the organoid. Overall, Dox had a greater proportion of abnormal calcium transients. (**c**) Workflow for machine-learning classifier model development. (**d**) Confusion matrix results from the best-performing model. (**e**) ROC curve for best-performing model, AUC = 0.98.

A variety of abnormal calcium transients were observed in both control and Dox-treated cells (Figure 4a), which requires considerable time and effort for thorough examination. Expert classification yielded a total of 639 abnormal cells (58.4%), 291 within controls (49.6%), and 348 within Dox-treated cells (68.6%). Additionally, the proportion of abnormal cells increased within each spatial region further from the organoid in both groups (Figure 4b). We were able to train and test a support vector machine (SVM) model, a widely utilized supervised machine-learning model for classification, to classify calcium transients to facilitate the burden of this step in analysis (Figure 4c). We used a radial basis function for the kernel type, and the model performed best when tuning the C hyperparameter to a value of 1000. We evaluated the performance of our machine-learning classifier based on the confusion matrix, which depicts the number of correctly and incorrectly classified cells (Figure 4d). This allowed us to calculate values for sensitivity (correct abnormal classification), specificity (correct normal classification), and accuracy (total correct classifications), which were 94.7%, 89.7%, and 92.6%, respectively. We then plotted the sensitivity (true positive rate) against the false positive rate (1-specificity) at different classification thresholds to create a receiver-operating characteristic (ROC) curve to further demonstrate the accuracy of our model’s classification predictions. The area under the ROC curve value was 0.98, which indicates exceptional performance (Figure 4e).

### 3.5. Dysfunctional Contractility Reflects Dox-Related Calcium Abnormalities

**Figure 5.**
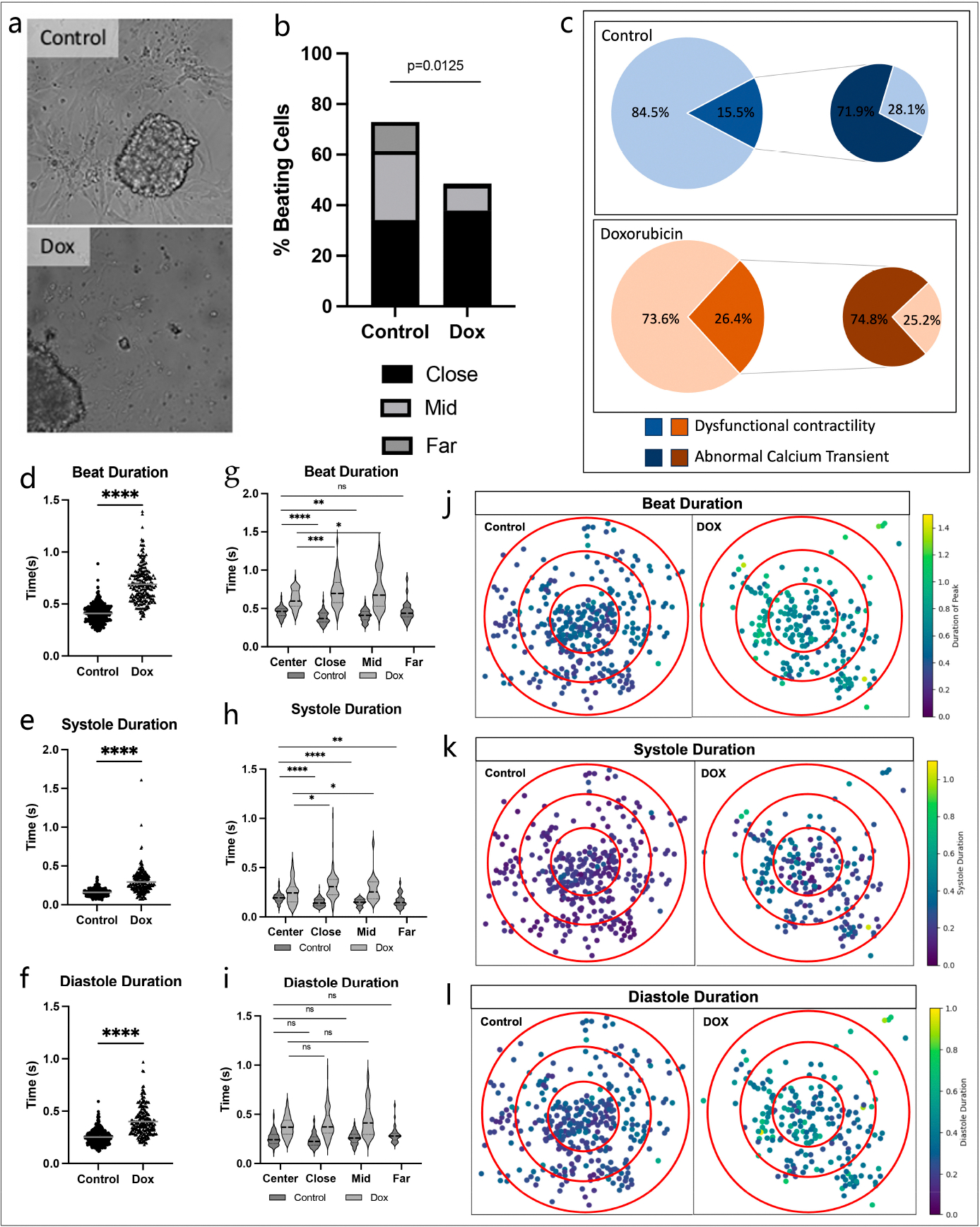
(**a**) Brightfield videos of control and Dox organoids and cells, can see in supplementary files. (**b**) Dox group had fewer beating cells, which were mostly concentrated in close region. (**c**) Dox had more cells with dysfunctional contractility. but in both groups, a similar percentage of dysfunctional beating can be attributed to abnormal calcium handling. (**d**) Total beat duration is prolonged in Dox group (Unpaired *t*-test, sn = 209–342. (**e**) Dox group has prolonged systole (Unpaired *t*-test, n = 209–342. (**f**) Dox group has prolonged diastole (Unpaired *t*-test, n = 209–342. (**g**–**i**) Beat, systole, and diastole durations have temporospatial changes (One-way ANOVA with Tukey post-hoc test, n = 23–144). (**j**–**l**) Heat map representation of temporospatial changes on a per-cell basis. (* *p* < 0.05, ** *p* < 0.01, *** *p* < 0.001, **** *p* < 0.0001, ns = non-specific).

To assess the effects of Dox and Dox-induced calcium overload on cellular function, we analyzed brightfield images of our model and found that defects in contractility properties correlated with observed calcium abnormalities. First, we found that Dox caused decreased cardiomyocyte function, as a lower proportion of cells per organoid had contractile motion (72.9 ± 5.24% vs. 48.5 ± 6.93%, *p* = 0.0125), and there were very few beating cells in the middle and far regions, indicating that this effect amplified with increased distance from the organoid (Figure 5b). We also found that among beating cells, 26.4% had dysfunctional contractility in the Dox group, compared to only 15.5% in the control group (Figure 5c). To assess the correlation between dysfunctional contractility and calcium dynamics, we analyzed the calcium transient data for the cells with abnormal beating patterns and found that both groups had similar proportions of abnormal calcium transients (71.9% in control vs. 74.8% in Dox group, Figure 5c). Total beat, systole, and diastole durations were significantly prolonged to a similar extent in our Dox-treated cells (total beat duration: 0.410 ± 0.005 s vs. 0.688 ± 0.012 s, *p* < 0.0001; systole: 0.159 ± 0.003 s vs. 0.293 ± 0.011 s, *p* < 0.0001; diastole: 0.252 ± 0.004 s vs. 0.400 ± 0.009 s, *p* < 0.0001; Figure 5d–f). In the control group, durations for systole and diastole varied across the different spatial regions, but there were no consistent findings of prolongation or shortening based on distance from the organoid. Conversely, we observed that total beat time and systole were longer in the surrounding cells than in the organoids in our Dox-treated cells (*p* < 0.05). Correlating with prolonged calcium reuptake, we also observed a consistent extension in diastole duration, although this did not reach statistical significance (Figure 5g–l). Differences in calcium fluorescence and contractility with and without Dox treatment are tabulated in Supplemental Table S1.

## 4. Discussion

Due to the need for improved assessment and prediction of cardiotoxicity during drug development and testing, we have developed a novel 2D/3D hybrid organoid model that uses optical imaging techniques such as fluorescence to study drug effects via calcium transients, contractility, and, most distinctly, signal propagation. We found that our novel 2D/3D hybrid organoid model accurately assessed the known cardiotoxic effects of Dox and, thus, presents a feasible method for evaluating drug cardiotoxicity on a cellular level in a high-throughput manner. To validate our model, we first assessed Dox cardiotoxicity and confirmed its known effects. As shown in the literature, calcium transient data demonstrated prolonged total peak durations, extended release and reuptake phases, reduced frequency, and decreased maximum rate of rise [12,13]. These results align with case studies of patients’ arrhythmias such as QT interval prolongation, extrasystoles, atrial fibrillation, and ventricular arrhythmias, all of which can in part be attributed to defects in calcium handling [13–16]. Previous studies in animal models and in 2D and 3D iPSC cultures have also demonstrated decreased heart rate, prolonged QT interval, and irregular beating pattern, as well as altered calcium dynamics, including decreased reuptake and increased concentration [9–11,17]. These data provide context and further validation of our results using calcium transients as a surrogate marker of cardiotoxicity.

Each of these previously used models, however, has drawbacks, namely related to consistency and feasibility. Our novel 2D/3D hybrid organoid model provides a more robust and optimal platform by optimally balancing the advantages and disadvantages of each. For example, results found in 2D cell culture have yielded contradictory effects, with some studies finding reduced beating rate and prolonged action potential duration, while others have reported increased frequency, no acute effects on beating, or a complete cessation of contractile motion [11,17,18]. Additionally, studies have shown that 2D-cultured cells lack the complexity seen in real tissues and have divergent transcriptomes and electromechanical properties, which offers an explanation for different toxicity responses observed in 2D versus 3D cell cultures [4,5,19–22]. Our model circumvents these discrepancies because the isolated single “2D” cells in our hybrid model are developed as part of the organoid, not as monolayers, thus preserving properties of true cardiomyocytes and cell–cell interactions, while still allowing for easy optical analysis without interfering adjacent signal. Overall, our hybrid model overcomes the challenges of other systems by presenting reliable, easy-to-interpret results in human cells that display the complexity and structure of cardiomyocytes.

Furthermore, what distinguishes our hybrid model from others is the dispersion of cells around the beating organoid. This allows us to study signal propagation and how the signal changes from its origin on a per cell basis. This is important because a major contributor to arrhythmias is conduction velocity, which is hypothesized to be impaired in Dox cardiotoxicity [6,23]. Our results demonstrated diastole prolongation with increased distance from the beating organoid, suggesting that Dox cardiotoxicity amplifies across a signal. This can contribute to arrhythmias due to calcium overload, impacting the initiation of the next action potential, which can predispose cells to altered signals and arrhythmias. Correlative findings have also been previously reported in human patients, 2D and 3D cell cultures, and in animal models, which have shown increased QT dispersion, increased field potential duration, increased dispersion of repolarization, and reduced and heterogenous conduction velocity [11,24–28]. Although these findings report different metrics, they converge on the underlying observation that signal propagation is not uniform throughout, and such signal dispersion and electrical heterogeneity with nonuniform repolarization and refractoriness can induce arrhythmias. Mechanistic investigations have supported these conclusions, demonstrating that Dox and calcium overload impact connexin-43, the main gap junction protein found in cardiac tissue [29–32]. Specifically, Dox has been found to impair the expression and function of connexin-43, which can lead to conduction velocity slowing and heterogeneity, while calcium overload has been shown to exacerbate conduction slowing through down-regulated gap junction communication and electrical uncoupling [30–36]. Thus, there are several mechanisms that contribute to cell–cell communication and offer explanations for the impaired signal propagation with increasing distance from the signal origin demonstrated in our model.

Additionally, our results reflect what has been elucidated from molecular studies of Dox cardiotoxicity and abnormal calcium handling. Firstly, Dox is known to cause a pro-apoptotic environment and decrease cell viability, which explains why we have overall fewer cells in our Dox-treated models [11,12,31] The prolonged time for calcium reuptake and the overall increased levels of calcium found in our study are reflective of previous work identifying dysfunctional SERCA2a proteins as a cause for impaired calcium release and reuptake in Dox cardiotoxicity [9,32,37,38]. In addition to direct effects seen on SERCA2a expression, Dox has been shown to increase reactive oxygen species. These reactive oxygen species can alter SERCA2a activity and cause diastolic calcium leak, contractile dysfunction, and impaired cardiomyocyte relaxation, which we have also observed in our model [12,39,40]. Impaired SERCA2a expression and function are a proposed mechanism for abnormal contractility and have also been seen in heart failure, arrhythmias, and ischemic heart disease, and therefore are a likely contributor to Dox cardiotoxicity [41–43].

Considering the tight control of excitation–contraction coupling, it is not surprising that our Dox-treated cells also display impaired contractility that is reflective of the effects seen on calcium transients. Previous studies have elucidated that the calcium transient decay rate closely mirrors myocyte contraction. This implies that decreased calcium reuptake and subsequent elevated intracellular calcium levels during diastole play a significant role in impaired and delayed relaxation [44]. Specifically in Dox models, depressed contractility and impaired relaxation have been attributed to altered sarcoplasmic reticulum calcium release and uptake [45,46]. Furthermore, Dox and its associated calcium overload have been shown to reduce contractile structures and impair their function due to sarcomeric disarray and myofibril deterioration [47–50]. This provides an explanation for why some of our cells with dysfunctional contractility did not have calcium abnormalities. On the other hand, studies have shown that calcium transients become abnormal significantly earlier than contractility defects arise, explaining why not all cells with abnormal calcium have abnormal beating [51]. Therefore, our model provides additional insight into the excitation–contraction coupling impairment in Dox cardiotoxicity and can clarify if it is attributable to calcium, cellular architecture, or both.

Given that many of the detrimental effects caused by Dox can be attributed to abnormal calcium handling, this study emphasizes the need to correctly detect normal versus abnormal calcium transients. When done manually, this process is burdensome, inefficient, and prone to human error due to borderline signals, output noise, subjectivity, and expertise. Therefore, we additionally developed a machine-learning classifier for assessing normality. Previous studies have shown the ability of various machine-learning methods, including SVM, to successfully classify cells based on calcium transient data. This has been done to assess impaired relaxation, predict the mechanistic action of cardioactive drugs, classify different cardiac diseases, and categorize normal versus abnormal calcium transients [52–55]. These models have all had impressive accuracy, ranging from 80–88%, and our models had comparable results with an accuracy improved to 92.6%. Because irregularities may relate not only to transient morphology but also to signal phase, and because cells may occasionally have a singular abnormal signal amongst normal transients and vice versa, we classified calcium transients on a whole-cell basis. We based it on averaged parameters, rather than a single peak basis, to obtain the most accurate classification of each cell to assess the overall cardiotoxic burden. Additionally, since both control and Dox-treated cells had calcium transients of normal and abnormal morphology, we trained and tested our model to classify calcium transients regardless of drug-treatment status in order to capture underlying patterns of calcium abnormality in an unbiased manner, ensuring it was not solely classifying based on drug status. This improves its generalizability among cardiomyocytes with different cardiotoxicities, which will enhance its ability to screen a variety of drugs in a high-throughput manner.

## 5. Limitations

Despite overcoming the challenges of previously utilized model systems for assessing drug cardiotoxicity, our study is subject to the following limitations. We focused solely on doxorubicin and assessed only one concentration at a single time point. Future studies employing our model with a broader range of compounds at a variety of concentrations at different time points will provide further validation and proof of utility. Given our model’s ability to screen drugs in a high-throughput manner, this can easily be achieved. Additionally, we imaged calcium transients and contractility consecutively, as opposed to simultaneously, which limited our ability to observe interactions that may have occurred concurrently. Assessment of these two parameters in conjunction can be done with this model, with the proper equipment, and would shed further light on a drug’s impact on excitation–contraction coupling.

## 6. Conclusions

In conclusion, our 2D/3D hybrid organoid model has the potential to offer a novel, high-throughput, low-cost, and effective drug screening tool that can be utilized to assess several factors impacting cardiac health, such as calcium transients, contractility, and signal propagation. Given that most cardiotoxic effects relate to pro-arrhythmia, our model is well-suited to study most drugs. It additionally allows for studying contractility in association with electrical effects. The isolation of single cells can also allow for staining and assessment of other structures impacted in drug-related cardiotoxicity, such as mitochondria, cellular architecture, and alpha actinin. Furthermore, it has the potential to provide personalized, precision medicine by utilizing iPSCs directly from patients to study how they may uniquely react to potential drug candidates.

## Supplementary Material

Supplementary file movies

**Supplementary Materials:** The following supporting information can be downloaded at https://www.mdpi.com/article/10.3390/therapeutics2030011/s1: Table S1: Relationship between adjacent spatial zones with respect to parameters accessed including peak duration, calcium release duration, calcium reuptake duration, and max rate of rise for calcium transients and the corresponding contractility characteristics of beat duration, systole duration, and diastole duration. Change in color shade as compared to the color shade above represents a statistical difference in the measured parameter. Video S1: 2D sheet of iPSC-CMs beating in Figure 1a. Video S2: 3D organoid beating in Figure 1a. Video S3: Representative videos of control organoids and cells with calcium-sensitive Rhod-2 dye in Figure 2b. Video S4: Representative videos of Dox organoids and cells with calcium-sensitive Rhod-2 dye in Figure 2b. Video S5: Brightfield videos of control organoids and cells in Figure 5a; Video S6: Brightfield videos of Dox organoids and cells in Figure 5a.

## Data Availability

The data reported in this study are available and can be requested by contacting the corresponding author.
