# Supplementary material for: Novel 2D/3D Hybrid Organoid System for High-Throughput Drug Screening in iPSC Cardiomyocytes
Source: Therapeutics (Basel). Author manuscript; Available in PMC 2026 Feb 12. (PMC12893397; doi:10.3390/therapeutics2030011)
Supplement: Supplementary file movies [file NIHMS2132039-supplement-Supplementary_file_movies.zip › Supplementary Table S1.pdf]

Calcium transients			Contractility	
Peak Duration			Beat Duration	
	Control	Dox+	Control	Dox+
Center				
Close				
Mid				
Far				
Calcium Release Duration			Systole Duration	
Center				
Close				
Mid				
Far				
Calcium Reuptake Duration			Diastole Duration	
Center				
Close				
Mid				
Far				
Max Rate of Rise				
Center				
Close				
Mid				
Far				

Supplementary Table 1: *Relationship between adjacent spatial zones with respect to parameters accessed including peak duration, calcium release duration, calcium reuptake duration and max rate of rise for calcium transients and the corresponding contractility characteristics of beat duration, systole duration and diastole duration. Change in color shade as compared to the color shade above represents a statistical difference in measured parameter.*
